# A Practical Risk-Based Model for Early Warning of Seafarer Errors Using Integrated Bayesian Network and SPAR-H

**DOI:** 10.3390/ijerph191610271

**Published:** 2022-08-18

**Authors:** Wenjun Zhang, Xiangkun Meng, Xue Yang, Hongguang Lyu, Xiang-Yu Zhou, Qingwu Wang

**Affiliations:** Navigation College, Dalian Maritime University, No. 1, Linghai Road, Dalian 116026, China

**Keywords:** Bayesian network, risk-based early warning, performance-shaping factor, SPAR-H, unsafe crew acts

## Abstract

**Highlights:**

- A BN version of the SPAR-H model is used to predict and warn of human errors to avoid maritime accidents and ensure the safety of seafarers.
- Performance-shaping factors (PSFs) are used as factors contributing to unsafe crew acts (UCAs).
- The conditional probabilities quantitatively describe the relationships among PSFs, UCAs, and human errors.
- The method offers a point for translating the research model into practical application.

**Abstract:**

Unsafe crew acts (UCAs) related to human errors are the main contributors to maritime accidents. The prediction of unsafe crew acts will provide an early warning for maritime accidents, which is significant to shipping companies. However, there exist gaps between the prediction models developed by researchers and those adopted by practitioners in human risk analysis (HRA) of the maritime industry. In addition, most research regarding human factors of maritime safety has concentrated on hazard identification or accident analysis, but not on early warning of UCAs. This paper proposes a Bayesian network (BN) version of the Standardized Plant Analysis Risk–Human Reliability Analysis (SPAR-H) method to predict the probability of seafarers’ unsafe acts. After the identification of performance-shaping factors (PSFs) that influence seafarers’ unsafe acts during navigation, the developed prediction model, which integrates the practicability of SPAR-H and the forward and backward inference functions of BN, is adopted to evaluate the probabilistic risk of unsafe acts and PSFs. The model can also be used when the available information is insufficient. Case studies demonstrate the practicability of the model in quantitatively predicting unsafe crew acts. The method allows evaluating whether a seafarer is capable of fulfilling their responsibility and providing an early warning for decision-makers, thereby avoiding human errors and sequentially preventing maritime accidents. The method can also be considered as a starting point for applying the efforts of HRA researchers to the real world for practitioners.

## 1. Introduction

Maritime transportation is characterized as one of the high-risk industries [1,2]. For this reason, the maritime industry has implemented management and technical measures aiming at improving its safety level [3]. Although progressive automation, new technologies, and improved safety measures have brought an improvement in maritime transportation safety, maritime accidents remain a major concern. Maritime accidents may cause casualties, financial losses, and/or environmental pollution [4]. The International Maritime Organization (IMO) recognized that human errors are the leading causes of maritime accidents [5]. The European Union investigation bodies launched 833 investigations from 2014 to 2019 and found that, of 1801 accidents, 54% of them were attributed to human factors [6]. Hence, assessing unsafe crew acts (UCAs) related to human errors plays a significant role in preventing maritime accidents.

**Figure 1:**
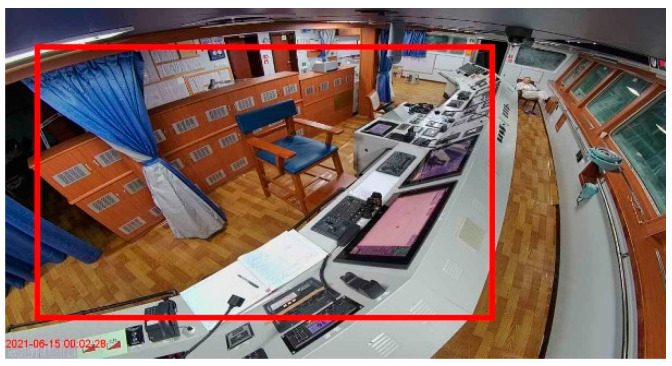
Real-time monitoring of leaving one’s post.

**Figure 2:**
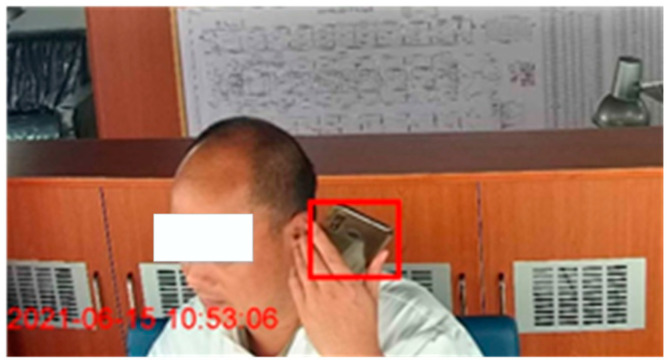
Real-time monitoring of the use of a cellphone.

Some UCAs, such as procedure violations, can be detected using technical means and predicted by identifying and assessing factors resulting in human errors. The procedure violations include the watchkeeper falling asleep, leaving one’s post, use of drugs or alcohol, use of cellphones, etc. These unsafe acts can be detected by utilizing data derived from multi-type sensors, including on-body wearable sensors, cameras, and microphones placed on board. The crew member can be warned in a timely manner so that they can work safely. Detection models can be built to continuously monitor procedure violations through the above detection techniques (Figure 1 and Figure 2) [7].

**Figure 3:**
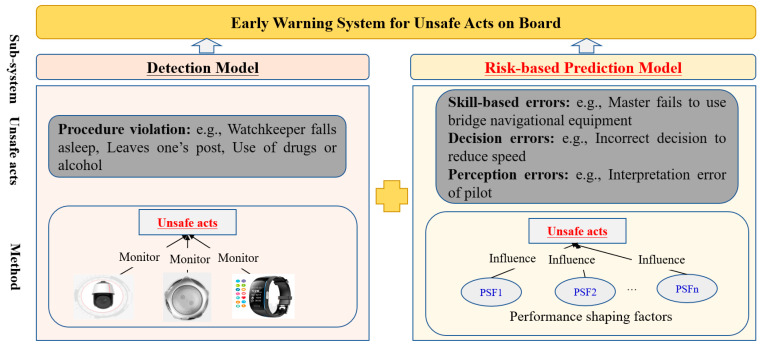
Detection and prediction of unsafe crew acts.

The procedures, training, task characteristics, work conditions, and work processes must be evaluated to make them compatible with seafarers’ competence. However, it is difficult to directly detect unsafe acts related to skill-based errors (e.g., a master failing to use bridge navigational equipment or being unskilled), decision errors (e.g., incorrect decision to reduce speed or incorrect decision-making for collision prevention), or perceptual errors (e.g., interpretation error of a pilot), which are also noteworthy factors contributing to the degradation of human performance. To improve human reliability and prevent maritime accidents, there is a need to figure out how these factors contribute to UCAs. The recognition of performance-shaping factors (PSFs) is one of the phases of unsafe acts assessment [8]. These errors can be predicted and warned of early by using performance-shaping factors (PSFs). PSFs cover multiple factors, such as personal characteristics, task characteristics, and work conditions. The hybrid model for assessing UCAs includes a detection model and a risk-based prediction model, as shown in Figure 3. This study focuses on using PSFs to predict unsafe acts that cannot be directly detected.

Focusing on the drawbacks of traditional methods, this paper developed a BN version of the SPAR-H (Standardized Plant Analysis Risk–Human Reliability Analysis) model with the integrated advantages of the practicability of SPAR-H and modeling uncertainty of BN. SPAR-H is primarily applied to select and quantify PSFs contributing to UCAs. Since BN can represent the causal relationships between factors using joint probability distributions, SPAR-H is then transformed into a BN to provide an integrated and more accurate model to predict and warn of UCAs. Compared with most research regarding human factors focusing on hazard identification or accident analysis, the present study offers an objective and risk-based way to calculate the probabilities of seafarer errors, and thus enable practitioners to judge whether a seafarer is fit for duty or not.

The remainder of this paper is organized as follows: Section 2 briefly reviews the literature on the assessment of UCAs and human errors in the maritime industry. Section 3 describes the approaches, including the BN, the SPAR-H, and their integration. Section 4 uses a case study to demonstrate the applicability of the developed model. Section 5 concludes this work.

## 2. Literature Review

UCAs occur when human capabilities cannot satisfy system demands [9]. These unsafe acts may further lead to accidents, deaths, or injuries. Human behaviors have gained continuously more significance in the formal safety assessment (FSA) on ship accidents. The Human Factor Analysis and Classification System (HFACS) has been used to analyze maritime incidents or accidents triggered by unsafe acts [10]. Examples include general analysis [11] and specific ones, e.g., ship collisions [3], passenger vessel accidents [12,13], and unmanned vessels’ safety [14]. Nevertheless, HFACS only gives a qualitative account of human factors and remains inadequate for probabilistic risk analysis (PRA).

The FSA of navigation used in the IMO’s role includes the PRA of specific accidental events. PRA, which involves identifying what can go wrong, determining how likely it is, and supporting operational decision-making, has attracted widespread concern in recent projects. In the field of marine transportation, some researchers presented the state-of-the-art of PRA. Among the exemplary articles, Goerlandt and Montewka (2015) discussed the risk definition and assessment approach for maritime transportation [15]; Chen et al. (2019) reviewed the extensive literature on the PRA of ship collisions from the perspective of methods and applications [16]. They concluded that uncertainties and limitations in data and information are still problems for acquiring reliable estimations of causation probabilities induced by UCAs in collision accidents.

PRA needs to model the complex dependencies between humans and systems. Bayesian Network (BN), considering the relationships of PSFs with a robust reasoning mechanism [17], has become an increasingly popular approach for PRA. Some researchers have used BN to address risk-oriented issues related to maritime safety. For example, Hänninen et al. (2014) modeled the factors influencing maritime safety and their dependencies with BN [18]. Utne et al. (2020) integrated BN with systems theoretic process analysis to model the online risk of autonomous ships [19]. Zhang et al. (2020) used BN to evaluate the nonfatal injury risks of seafarers based on data-driven technology and an empirical survey [20]. To estimate collision probabilities, Sotiralis et al. (2016) presented a BN-based risk model that incorporates human factors into modeling ship operation risks [21]. The present study also focuses on developing a risk-based prediction model that integrates leading indicators of human errors using BN. In a BN model, the human error probability (HEP) is recognized as the conditional probability of the PSFs, which can be discretized into states or levels.

The contributing factors to human errors, PSFs, are environmental, personal, or task-oriented factors and can be identified and managed [22]. PSFs resulting in unsafe acts in maritime accidents include fatigue, stress, lack of competence, inadequate training or experience, poor communication, bad safety culture or work procedures, and lack of situational assessment [23,24]. As a result, if these contributing PSFs are not controlled properly, human errors may occur and subsequently lead to major accidents. In the majority of cases, maritime accidents regarding human errors are induced by one of the above causes, or a combination of them. Hence, avoiding the bad levels of these PSFs will prevent maritime accidents from occurring, or reduce their probabilities.

Most research regarding human factors of maritime safety has concentrated on hazard identification or accident analysis [21,23,25], but not on the early warning of UCAs. Although many human error analysis methods have been developed by researchers, there is still a gap compared with the application requirements of practitioners. In high-reliability organizations, the SPAR-H method has been widely used due to its simplicity and practicability. SPAR-H was first used by U.S. nuclear power plants to assess HEPs [26], and is now also used in the petrochemical industry, such as the chemical plants [27], oil storage companies [28], and the deepwater drilling process [29]. Furthermore, Gould et al. (2012) considered that SPAR-H was easier to use in comparison with other human risk analysis (HRA) models, such as HFACS [30]. Steijn et al. (2020) also pointed out that SPAR-H is the most practical method for HRA. SPAR-H also has its limitations [28]. Paltrinieri et al. (2016) considered that SPAR-H needs a detailed task analysis by practitioners in specific scenarios [31]. SPAR-H allows reasoning about the HEP, but it cannot reason about PSFs. SPAR-H provides a point estimate for HEPs under the condition of all known states of PSFs; that is, the practitioner cannot reason about the HEP if the information related to PSFs is incomplete. In practice, however, there exists uncertain information in its application, and it is hard to completely know the states of all PSFs. Hence, there is a need for a more substantiated method to bridge the aforementioned gap. The purpose of this paper is, therefore, to predict UCAs and thus enable decision-makers to take proactive measures to avoid human errors, thereby reducing the risk of maritime accidents. 

## 3. Materials and Methods

### 3.1. Bayesian Network

BN provides an effective way to deal with risk and reliability assessment of complex systems with deterministic and uncertain information and dependencies. Based on the graph theory, BN consists of both qualitative and quantitative elements [32]. The former is modeled by a topology relationship, while the latter is represented by node state probabilities and conditional probability tables (CPTs) used for describing the strength of node dependencies [33,34]. 

Assume that there are *n* nodes in a BN. When the prior node probabilities and CPTs are known, the joint probability of variables *U* and *P*(*U*) can be expressed as [35]:

(1) $$P\left(U\right)=\prod_{i=1}^{n}P\left(X_{i}\left|Pa\left(X_{i}\right)\right.\right)$$

where *Pa* (*X_i_*) is the parent node of node *X_i_* and *U* = (*X*_1_, …, *X_n_*). According to Equation (1), the probability of *X_i_* is formulated as:

(2) $$P\left(X_{i}\right)=\sum_{U\backslash X_{i}}P(U)$$

where the summation contains all the variables of *U*, excluding *X_i_*. 

Besides causal reasoning, Bayes’ theorem also allows evidential reasoning, i.e., backward reasoning from human errors to PSFs, with which the practitioner can identify the most contributing influencing factors. When new evidence *E* is introduced, BN will be able to update the prior probabilities into posterior probabilities *P*(*U*|*E*) [36]:

(3) $$P\left(U|E\right)=P(U,E)/P(E)=P(U,E)/\sum_{U}P(U,E)$$

### 3.2. SPAR-H

SPAR-H provides a point estimate for UCAs by analyzing a specific task. The method is implemented in the following steps [37]:

#### 3.2.1. Identifying the Type of Task

SPAR-H uses multipliers to amend the base or nominal HEP (NHEP). The crew members usually execute two categories of tasks: diagnoses and actions. The NHEP is assigned as 0.01 for diagnoses and 0.001 for actions [37]. The present study aims to explore the HEP during navigation, so it is mainly about the action tasks, and the NHEP is 0.001.

#### 3.2.2. Determining the Multipliers According to PSF Levels

SPAR-H considers the influences of eight PSFs on human performances. All PSFs are rated on a specific level, e.g., good, normal, or poor, which depends on the task analysis [28]. Each level of the PSF corresponds to a multiplier, as listed in Table 1 [38].

#### 3.2.3. Calculating HEP

SPAR-H calculates the *HEP* with two equations, and the selection of equations is up to the number of negative PSFs. If there are fewer than three negative PSFs, Equation (4) is used; otherwise, Equation (5) is used. In the two equations, *NHEP* is 0.001, and *S_i_* represents the multiplier in Table 1 [39].

(4) $$HEP=NHEP\cdot \prod_{1}^{8}S_{i}$$

(5) $$HEP=\frac{NHEP\cdot \prod_{1}^{8}S_{i}}{NHEP\cdot \left(\prod_{1}^{8}S_{i}-1\right)+1}$$

### 3.3. Method Integration: BN Version of SPAR-H

A hybrid risk-based prediction model integrating SPAR-H and BN was developed to assess the notional probability of human performance and accordingly identify the critical factors. The integrated method is as follows.

#### 3.3.1. Developing BN for Unsafe Crew Acts

**Figure 4:**
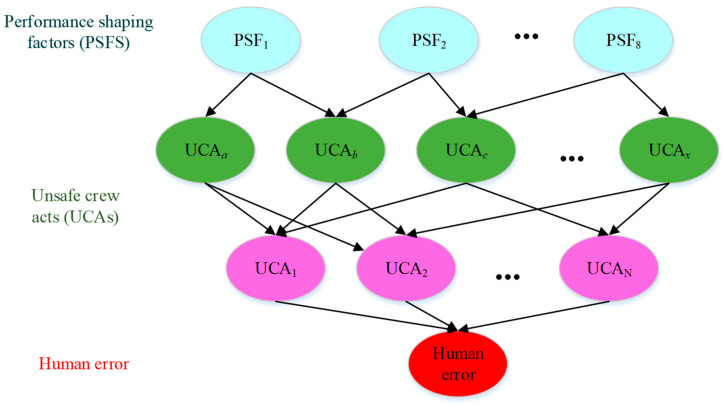
BN-based causal model.

The UCAs causing human errors and the PSFs leading to UCAs are identified. A BN, as shown in Figure 4, models the causal relationships among human errors, UCAs, and the PSFs listed in Table 1. The bottom layer is human error. The UCAs, which determine the HEP, constitute the intermediate layer. The PSFs form the top layer. The relationships among the layers can be obtained by reference to the literature and expert opinions.

#### 3.3.2. Quantifying the BN Model

Every node in the top layer is assigned prior probability values for its possible levels, which will be used for inference when information is insufficient. The conditional probabilities can be determined from the available information, expert opinions, deterministic relationships, or their combination. To calculate the HEP, the following three probabilities need to be determined: *P*(*PSF_i_*), *P*(*UCA_i_*|*pa*(*PSF_i_*)), and *P*(*HEP*|*pa*(*UCA_i_*)).

***P*(*PSF_i_*)**: As few human-related data are available, *P*(*PSF_x_*) can be obtained by reference to the literature, historical data, or expert opinions. 

***P*(*UCA_i_*|*pa*(*PSF_i_*))**: Map the main UCAs causing human errors to the PSFs of SPAR-H. Each UCA has a CPT that describes the effects of combinations of PSFs on the node. *P*(*UCA_i_*| *pa*(*PSF_i_*)) is calculated using Equation (4) or Equation (5). 

***P*(*HEP*|*pa*(*UCA_i_*)):** The size of CPTs will increase exponentially with the increment of parent nodes (Yu et al., 2021) [40]. Noisy-OR logic can deal with the deficiency and is used to describe the relationships between UCAs and the HEP. If one of the parent nodes *UCA_i_* occurs and other nodes are in positive states, the conditional probability of the child node human error can be calculated as:

(6) $$P_{UCA}{}_{{}_{i}}=P(HEP=1|\bar{UCA_{1}},\;\bar{UCA_{2}},\;\cdots ,\;UCA_{i},\;\bar{UCA_{i+1}},\;\cdots ,\;\bar{UCA_{N}})$$

Based on the Noisy-OR gate model, the *HEP* can be determined by

(7) $$P(HEP=1)=1-\prod_{i=1}^{N}\left(1-P_{UCA}{}_{{}_{i}}\right)$$

## 4. Case Study

This section demonstrates the method through a case study where a BN version of the SPAR-H model is developed, allowing for the probabilistic cause–consequence relationships among PSFs, UCAs, and HEP to be determined.

### 4.1. Case Description

The investigation into maritime accidents has demonstrated that most UCAs occur during tasks of supervision, navigation, and monitoring. These tasks are usually executed by Officers of the Watch (OOWs) on the bridge [41]. Hence, the bridge is one of the focal points to efficiently sustain operations during navigation, where the OOWs need to execute routine tasks, identify abnormalities, and cope with unsafe situations quickly using complex interfaces. Although the automatic degree is increasing on the bridge, OOWs, as the last safety barrier, still play vital roles in dealing with abnormal incidents. One of the causes of maritime accidents is that OOWs are incapable of predicting all potential accidents and cannot take pre-defined measures for all contingencies [21]. 

### 4.2. Qualitative Analysis: Mapping PSFs and UCAs

The UCAs and PSFs can be derived from a literature review, historical maritime accident reports, and questionnaires distributed to experienced seafarers. Most of them are latent factors and it is difficult to measure the extent to which they influence the probabilities of the OOWs’ performance. The PSFs contributing to UCAs include work conditions, the external environment, procedures, technology, training, organization, and individual factors (e.g., fatigue, experience, and mental state) [8,42,43].

#### 4.2.1. OOW’s Task Analysis

The starting point for developing the causal model is to analyze the tasks performed by the OOW. The main task of the OOW is to operate the ship safely and properly. The core of task analysis is the OOW’s cognitive process and the task context where the OOW makes errors. The focus is on coding the context leading to OOW’s errors and analyzing the PSFs that may influence the OOW in an erroneous act.

Sotiralis et al. (2016) divided the PSFs of the TRACEr (Technique for the Retrospective and predictive Analysis of Cognitive Errors) taxonomy into five categories: personal factors, communication/information, internal/external environment, organizational factors, and training/competence [21]. The adapted PSFs apply to the maritime context and are used to analyze accident reports. Erden and Akyuz (2021) adopted seven PSFs to describe human performance during the operation of container ships and calculate the HEP [44]. They divided the PSFs into external factors and internal factors. The former includes stress, limited time, complexity, and safety culture, while the latter involves experience, training, and fatigue.

**Figure 5:**
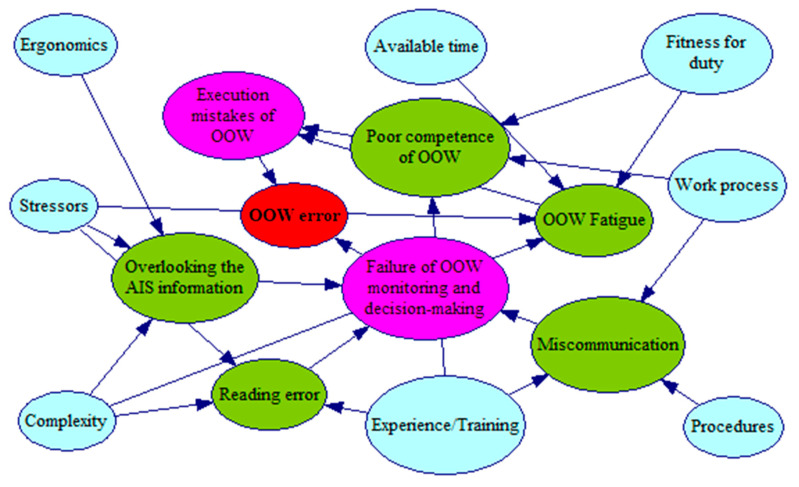
BN model of the OOW’s error.

The OOW performs the navigational tasks of monitoring the abnormal environment, making decisions, communicating with the bridge team and the outside, and operating the ship. UCAs such as overlooking the key alarm, reading error, lack of communication, fatigue, or poor competence may lead to these tasks failing during the execution process. Following the practices of the above studies, this study uses the standard PSFs as basic nodes to describe the factors leading to UCAs. The detailed descriptions of PSFs and UCAs are listed in Table 2, and Figure 5 depicts a graphical representation of the causal relationships among these nodes using the software GeNIe. 

#### 4.2.2. BN Structure

The BN model was developed consisting of a set of nodes representing PSFs, UCAs, and the OOW error. The end node describes the OOW error, while the nodes related to PSFs constitute the top layers and reflect the states or levels of factors that contribute to UCAs in the intermediate layers. The intermediate nodes mainly referred to the articles [21,23,24,25].

The developed model aims to calculate OOW errors due to human-related variables. The node states are assigned as follows: the states of PSFs are set based on their levels (e.g., good, normal, or poor), the UCAs are assigned a binary state (i.e., yes/no), and the end node also has two states: yes or no.

The probabilities of PSF nodes can be collected from the literature, historical data, or expert opinions. In fact, it will be best to gather data from historical reports about PSFs related to human errors in the maritime industry. This study emphasizes the feasibility of the developed model with the case study. Hence, the case study directly uses data derived from NUREG/CR-6949 as the prior probabilities of nodes related to PSFs [45]. The CPTs of the intermediate nodes related to UCAs, which have the parent nodes of PSFs, are assigned using the results calculated with Equations (4) and (5). 

Table 3 takes the node of “Poor competence of OOW” as an example and lists the calculated results. The node has three parent nodes: Experience/training, Fitness for duty, and Work processes. In the case of “Good”, “Normal”, and “Good” states of the three parent nodes, the number of negative states is zero and the conditional probability calculated by Equation (4) is 0.000125. In the case of “Poor”, “Unfit”, and “Poor” states, the number of negative states is three and the conditional probability calculated by Equation (5) is 0.2016. Other conditional probabilities can also be calculated in the same way.

The CPTs of other UCAs are determined using an OR logic gate. If the number of parent nodes of UCAs and the end node, the Noisy-OR logic can be used to quantify the CPTs among them. 

### 4.3. Results and Discussion

The developed model can perform both forward (causal) and backward (evidential) reasoning by calculating the prior and posterior probabilities of PSFs, UCAs, and OOW errors. Using causal reasoning, the prior probability of OOW errors calculated with the assignment of node probabilities and CPTs is 0.029. With this result, the practitioner can determine whether the OOW is fitting for the task compared with the acceptable risk of human errors. 

**Figure 6:**
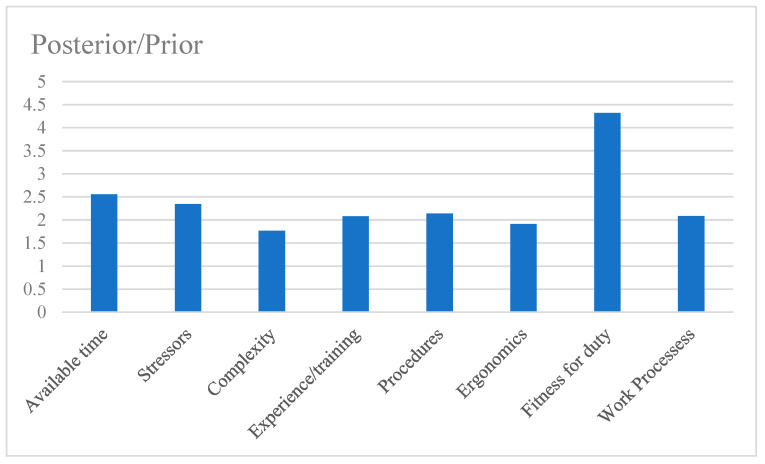
Posterior/prior values of the negative PSFs.

The posterior probabilities can be calculated by setting evidence in given states of the chosen nodes. For example, when the state of OOW error is set as “yes”, the posterior probabilities of all nodes can be obtained, as listed in Table 4. A bar graph is drawn with the posterior/prior value of the negative state of each PSF, which illustrates the individual contribution of each PSF to human error, as shown in Figure 6.

A large posterior/prior value means a great influence of PSFs on the OOW error. In light of the prior and posterior probabilities of the case study, the results indicate that “Fitness for duty” is the most contributory PSF to the OOW error (Figure 6). This PSF can be recognized as the most vulnerable root factor among all of the PSFs in the case study. It can be explained that degraded fitness may result in the fatigue of the OOW, who faces excessive tasks and confined space conditions during work. Fatigue causes a decline in alertness, safety awareness, and motivation, increases reaction time, and thus increases the OOWs’ error probabilities. “Available time” is the second-most-vulnerable root factor for OOW errors in the case study. Inadequate time, such as a crew member being oblivious to the coming of another ship or discovering it too late, will increase the error probability of properly executing the collision-avoidance task. 

The results depend on the assignment of prior probabilities of PSFs. For example, if the prior probability of the “Expensive time” state of the “Available time” node is changed to 0.259, the value of the OOW error probability will become 0.023, less than the value in the original case study. Accordingly, the posterior probabilities will also change, and the practitioner can then adjust the human risk control measures.

The developed method functionality provides the practitioner with early warning and diagnostic insight into the influencing factors of human errors. Decision-makers can then take emergent and appropriate measures to prevent unsafe acts under different performance contexts.

## 5. Conclusions

Ensuring the safety of seafarers during maritime transportation is one of the main concerns of maritime authorities. Given this, HRA is an important consideration in predicting human errors (unsafe acts) and preventing casualties from occurring. This study emphasizes the performance-shaping factors of UCAs and predicts the human error probabilities. A hybrid model integrating SPAR-H with BN was developed due to the practicability of the former and the reasoning function of the latter.

HRA methods require a robust technical basis and need to describe relationships among factors. BN is a robust model that allows the integration of information from various sources and provides a mechanism for causal and evidential reasoning. HRA methods should also be compatible with the needs of HRA practitioners. SPAR-H, as the most practical HRA model, is widely used by practitioners. The BN version of the SPAR-H model is complementary, thereby bridging gaps between HRA research and practice. 

Compared with most research regarding human factors of maritime safety focusing on hazard identification or accident analysis, the present study aims to predict UCAs, and thus enable decision-makers to take proactive measures to avoid human errors. The case study demonstrates that the developed model can be effectively applied for the calculation of HEP. Determining the risks related to the most vulnerable human error factors during maritime transportation will contribute to improving seafarer safety and avoiding accidents. Hence, preventive measures can be taken for tasks in which human errors may result in undesired accidents. 

Since both the understanding of PSFs and data sources of UCAs are continually evolving, additional PSFs can be added without altering the SPAR-H structure in future work. The proposed method can also be considered as a starting point for the continued development of practical risk-based early warning of human errors in maritime transportation.

## Figures and Tables

**Table 1:** SPAR-H PSFs, levels, and multipliers.

No.	PSF	PSF Level	Multiplier
1	Available time	Expansive time	0.1
		Normal time	1
		Barely adequate time	10
2	Stressors	Normal	1
		High	2
		Extreme	5
3	Complexity	Normal	1
		Moderately complex	2
		Highly complex	5
4	Experience/training	Good	0.5
		Normal	1
		Poor	5
5	Procedures	Normal	1
		Available, but poor	5
		Incomplete	20
		Not available	50
6	Ergonomics	Good	0.5
		Normal	1
		Poor	10
		Missing/misleading	50
7	Fitness for duty	Normal	1
		Degraded fitness	5
		Unfit	20
8	Work processes	Good	0.5
		Normal	1
		Poor	5

**Table 2:** Descriptions of PSFs and UCAs.

PSF	Description of UCA
Available time	This PSF involves whether the time is adequate to execute a task and whether the task execution satisfies the requirement of the process dynamics on board. In a collision case, inadequate time includes an event where a crew member of one ship is oblivious to the coming of another ship or discovers it too late.
Ergonomics	This PSF refers to features of the human–machine environment, including poor bridge design, the unreasonable layout of bridge instrumentation, or failure of instruments.
Stressors	This PSF accounts for mental conditions adversely influencing the performances of OOWs. Overwork, mental fatigue, insufficient incentive, and unreasonable working arrangement will lead to stressors.
Experience/Training	This PSF is related to crew members’ training time, quality and effect of training, education level, working seniority, etc.
Procedures	In the shipping industry, this PSF involves: (a) a safety policy that ensures the safe operation of ships; (b) the communication mechanism among shore and shipboard crews; (c) procedures for reporting incidents, near misses, and accidents; and (d) preparing for and responding to emergencies.
Work processes	This PSF refers to formal processes (operational tempo, time pressures, production quotas, incentive systems, schedules, etc.)
Complexity	Highly complex tasks refer to the situations in which crew members have no knowledge, aptitude, skill, or time to deal with them.
Fitness for duty	Unfitness for duty may occur when OOWs fail to prepare physically or mentally: violations of rest requirements (fatigue) or the use of drugs or alcohol.

**Table 3:** CPT of node “Poor competence of OOW”.

**Experience/Training**		Good								
**Fitness for Duty**		Normal			Degraded fitness			Unfit		
**Work Processes**		Good	Normal	Poor	Good	Normal	Poor	Good	Normal	Poor
**Poor Competence of OOW**	**Yes**	0.000125	0.00025	0.00125	0.000625	0.00125	0.00625	0.0025	0.005	0.025
	**No**	0.999875	0.99975	0.99875	0.999375	0.99875	0.99375	0.9975	0.995	0.975
**Experience/Training**		Normal								
**Fitness for Duty**		Normal			Degraded fitness			Unfit		
**Work Process**		Good	Normal	Poor	Good	Normal	Poor	Good	Normal	Poor
**Poor Competence of OOW**	**Yes**	0.00025	0.0005	0.0025	0.00125	0.0025	0.0125	0.005	0.01	0.05
	**No**	0.99975	0.9995	0.9975	0.99875	0.9975	0.9875	0.995	0.99	0.95
**Experience/Training**		Poor								
**Fitness for Duty**		Normal			Degraded fitness			Unfit		
**Work Processes**		Good	Normal	Poor	Good	Normal	Poor	Good	Normal	Poor
**Poor Competence of OOW**	**Yes**	0.00125	0.025	0.0125	0.00625	0.0125	0.0625	0.025	0.05	0.2016
	**No**	0.99875	0.975	0.9875	0.99375	0.9875	0.9375	0.975	0.95	0.7984

**Table 4:** Prior and posterior probability of every node.

Node	Node State	Prior Probability	Posterior Probability	Posterior/Prior
Available time	Expansive time	0.159	0.085	0.535
	Normal time	0.683	0.511	0.748
	Barely adequate time	0.158	0.404	**2.557**
Stressors	Normal	0.841	0.772	0.918
	High	0.136	0.174	1.279
	Extreme	0.023	0.054	**2.348**
Complexity	Normal	0.500	0.367	0.734
	Moderately complex	0.341	0.351	1.029
	Highly complex	0.159	0.281	**1.767**
Experience/training	Good	0.103	0.069	0.670
	Normal	0.714	0.550	0.770
	Poor	0.183	0.381	**2.082**
Procedures	Normal	0.450	0.355	0.789
	Available, but poor	0.300	0.273	0.91
	Incomplete	0.200	0.265	1.325
	Not available	0.050	0.107	**2.140**
Ergonomics	Good	0.158	0.149	0.943
	Normal	0.683	0.652	0.955
	Poor	0.136	0.154	1.132
	Missing/misleading	0.023	0.044	**1.913**
Fitness for duty	Normal	0.841	0.608	0.723
	Degraded fitness	0.109	0.175	1.606
	Unfit	0.050	0.216	**4.320**
Work processes	Good	0.158	0.119	0.753
	Normal	0.819	0.833	1.017
	Poor	0.023	0.048	**2.087**
Overlooking the AIS information	Yes	0.002	0.068	34.00
	No	0.998	0.932	0.934
Reading error	Yes	0.001	0.035	35.00
	No	0.999	0.965	0.966
Miscommunication	Yes	0.007	0.248	35.43
	No	0.993	0.752	0.757
OOW fatigue	Yes	0.008	0.498	**62.25**
	No	0.992	0.502	0.506
Poor competence	Yes	0.005	0.167	33.40
	No	0.995	0.833	0.837
Failure of OOW monitoring and decision-making	Yes	0.010	0.350	35.00
	No	0.990	0.650	0.657
Execution mistakes of OOW	Yes	0.013	0.660	50.77
	No	0.987	0.340	0.344
OOW error	Yes	0.029	1	34.48
	No	0.971	0	--
